# Cryptosporidiosis in Haiti: surprisingly low level of species diversity revealed by molecular characterization of *Cryptosporidium* oocysts from surface water and groundwater

**DOI:** 10.1051/parasite/2013045

**Published:** 2013-11-20

**Authors:** Céline Damiani, Ketty Balthazard-Accou, Elmyre Clervil, Aïssata Diallo, Cécilia Da Costa, Evens Emmanuel, Anne Totet, Patrice Agnamey

**Affiliations:** 1 Parasitology Laboratory – Mycology, Amiens University Hospital, Avenue Laënnec 80054 Amiens France; 2 University of Picardie Jules Verne, UFR Médecine 1 rue des Louvels 80037 Amiens Cedex 1 France; 3 Laboratory of Qualité de l’Eau et de l’Environnement, University Quiqueya BP 796 Port-au-Prince Haïti; 4 University of Picardie Jules Verne, UFR Pharmacie, Equipe théra, Laboratoire des Glucides-FRE-CNRS 3517 1 rue des Louvels 80037 Amiens Cedex 1 France

**Keywords:** Haiti, Water, Environmental Pollution, *Cryptosporidium* oocysts, Genotyping

## Abstract

The protozoan parasite *Cryptosporidium* sp. has emerged as one of the most important water contaminants, causing waterborne outbreaks of diarrhoeal diseases worldwide. In Haiti, cryptosporidiosis is a frequent cause of diarrhoea in children under the age of five years, HIV-infected individuals, and people living in low socioeconomic conditions, mainly due to the consumption of water or food polluted by *Cryptosporidium* oocysts. The aim of this study was to detect and identify *Cryptosporidium* oocysts present in 12 water samples collected in Port-au-Prince and 4 water samples collected in Cap Haïtien. Initial detection consisted of immunomagnetic separation – immunofluorescence assay (IMS-IFA), which was confirmed by nested PCR, targeting the most polymorphic region of the 18S rRNA gene in 15/16 samples. Genotyping was performed by PCR-restriction fragment length polymorphism (RFLP) analysis and DNA sequencing. Under our working conditions, neither nested PCR-RFLP nor direct DNA sequencing revealed the expected species diversity, as only *Cryptosporidium parvum* was identified in the water samples studied. This study highlights the difficulty of detecting mixed populations of *Cryptosporidium* species in environmental samples.

## Abstract (fr)

Le protozoaire parasite *Cryptosporidium* sp. apparaît comme un polluant hydrique majeur engendrant des épidémies diarrhéiques à travers le monde. En Haïti, la cryptosporidiose est responsable de la plupart des diarrhées chez les enfants âgés de moins de 5 ans, les patients VIH, les personnes vivant dans des conditions socioéconomiques difficiles, surtout suite à la consommation de nourritures et d’eau de boisson polluées par les oocystes de cryptosporidies. Le but de cette étude était de détecter et d’identifier des oocystes de cryptosporidies dans 12 prélèvements d’eau effectués à Port-au-Prince et 4 au Cap Haïtien. La méthode de détection initiale a été une séparation immunomagnétique couplée à l’immunofluorescence, confirmée par méthode de PCR nichée, utilisant des amorces spécifiques de la région la plus polymorphe du gène codant pour 18S rRNA, positive dans 15 des 16 prélèvements. Le génotypage a été réalisé par l’utilisation des enzymes de restriction et le séquençage direct de l’ADN. Dans les conditions de notre étude, ces deux méthodes n’ont pas révélé la diversité d’espèces attendue. En effet, seul *Cryptosporidium parvum* a été identifié dans tous les prélèvements étudiés. L’étude pointe la difficulté d’obtenir une bonne résolution de mélange d’espèces de cryptosporidies dans les échantillons environnementaux.

## Introduction

*Cryptosporidium* sp. is a protozoan parasite that causes diarrhoeal illness via faecal-oral transmission. It has an incubation period of up to 2 weeks. *Cryptosporidium parvum* was reported for the first time in 1907 in gastric crypts of a laboratory mouse [28]. It has been subsequently found in many other animals (chickens, turkeys, rats, guinea pigs, horses, etc.). The first human case was reported in 1976 [1]. *Cryptosporidium* has subsequently become a well-known cause of opportunistic infections among AIDS patients and is responsible for outbreaks of gastrointestinal disease including the memorable outbreak that occurred in Milwaukee, Wisconsin in 1993 involving 403,000 cryptosporidiosis infected individuals, 4,400 of whom were hospitalized and 69 died [20]. This outbreak was due to contamination of drinking water. Five years later, it was reported that residents of Sydney, Australia were infected by *Cryptosporidium* contamination of the drinking water supply from July to December 1998 [12]. *Cryptosporidium-*related diarrhoea is transient in immunocompetent individuals [13, 16, 29], while diarrhoea can become severe, chronic and life-threatening in immunocompromised patients, especially those infected by human immunodeficiency virus (HIV) [14, 19, 23]. No effective treatment of cryptosporidiosis is available at the present time. Cryptosporidiosis generally appears to be closely correlated with environmental causes, mainly in developing countries where most people live in inadequate hygiene conditions. In Haiti, cryptosporidiosis is a frequent cause of diarrhoea and is responsible for 17.5% of cases of acute diarrhoea in children under the age of 2 years and 30% of cases of chronic diarrhoea in HIV-infected patients [21, 22]. In Haiti, in the early 2000s, several environmental investigations were conducted in Port-au-Prince and the surrounding region, Les Cayes and Cap Haïtien in order to identify sources of human contamination of cryptosporidiosis. These surveys simply consisted of detecting *Cryptosporidium* oocysts in the environment by screening various types of water (surface water, groundwater, public water supplies) used by the population [3, 4, 24]. The main objective of these preliminary investigations was to evaluate the circulation level of *Cryptosporidium* oocysts in the Haiti environment. In the present study, the investigation comprised molecular characterization of environmental *Cryptosporidium* oocysts detected in order to identify *Cryptosporidium* species present in the survey zones.

## Materials and methods

### Collection of water samples

Cross-sectional surveys of domestic drinking water were conducted in Port-au-Prince and Cap Haïtien in October 2010 and January 2011, respectively. This study was therefore conducted after the earthquake in January 2010. In Port-au-Prince, water sampling was performed in the districts of Pétion Ville (No. 1; 2), Delmas (No. 3), Carrefour (No. 4) and Tabarre (No. 5–12). Regarding Cap Haïtien, water sampling was performed in the municipalities of Petite Anse (No. 13, 14) and Bande du Nord (No. 15, 16), (Table 1). To minimize cross-contamination in the field, new water sampling equipment (bucket, tumbler and funnel) was used at each site. A sample of at least 100 L of water was collected and immediately filtered using a polyethersulphone capsule (Environchek^®^, Pall Gelman, Saint Germain en Laye, France). Capsules were stored at 4 °C until the elution step.

**Table 1.** Characteristics of water sampling sites in Port-au-Prince and Cap Haïtien and results of *Cryptosporidium* oocysts detection and molecular characterization.

Site number	Sampling sites	Source of water samples	Number of litres filtered	Number of oocysts/100 L of water	Results of PCR detection	*Cryptosporidium* species identified	District/*Population served
*Port-au-Prince*							
1	Turgeau	Reservoir	340	67	+	*C. parvum*	Pétion-ville/342,451
2	Plaisance	Spring	255	33	+	*C. parvum*	
3	Delmas 71	Reservoir	170	53	+	*C. parvum*	Delmas/359,451
4	Dikini 63	Spring	272	145	+	*C. parvum*	Carrefour/465,019
5	Clercine F2	Bore	340	17	+	*C. parvum*	
6	Clercine F7	Bore	340	233	+	*C. parvum*	
7	Rivière Grise	Surface water	204	19	unsuccessful	unsuccessful	
8	Tabarre 25-F5	Bore	255	118	+	*C. parvum*	Tabarre/118,477
9	Tabarre 41-T4	Bore	170	20	+	*C. parvum*	
10	Tapage-T8	Bore	255	6	+	*C. parvum*	
11	Bureau Central CAMEP	Bore	340	32	+	*C. parvum*	
12	Tapage-T6	Bore	272	28	+	*C. parvum*	
*Cap Haïtien*							
13	Zone de Balan1	Bore	119	6,088	+	*C. parvum*	Petite Anse/98,373
14	Zone de Balan2	Bore	153	4,902	+	*C. parvum*	
15	En Haut Jean	Spring	170	5,640	+	*C. parvum*	Bande du Nord/21,157
16	Mandot	Reservoir	170	3,583	+	*C. parvum*	

* Inhabitants [15]; +: positive

### Purification of *Cryptosporidium* oocysts

Capsules were processed according to the Association Française de Normalisation (AFNOR) standard operating procedures [2]. Briefly, capsule filters were rinsed with 240 ml of a detergent elution buffer (phosphate-buffered Saline, pH 7.4 with 0.1% (v/v) Tween 80). Specimens were concentrated by centrifugation at 2000g for 20 min. The final sediment was suspended in double-distilled water to a final volume of around 5 ml. Any *Cryptosporidium* oocysts present were then purified using immunomagnetic beads coated with anti-*Cryptosporidium* monoclonal antibody (Dynabeads^®^, Dynal, Oslo, Norway) according to the manufacturer’s instructions. Following the immunomagnetic separation (IMS) procedure [5], about 50 μl of suspension was obtained and used for immunofluorescence assay (IFA) and molecular analysis.

### Detection and counting of *Cryptosporidium* oocysts

Twenty microlitres of suspension derived from the IMS procedure was placed on a glass slide and dried at room temperature. Slides were fixed in cold acetone (−20 °C) for 10 min and were then incubated for 30 min at 37 °C in a humid chamber with a 1:10 final dilution of a fluorescein isothiocyanate (FITC)-conjugated monoclonal antibody (MAb) directed against a *Cryptosporidium* wall antigen, which was selected because of its lack of cross-reactivity with other microorganisms (FITC-Cow MAb, Monofluokit *Cryptosporidium* ^®^, Bio-Rad, Marnes la Coquette, France). Slides were rinsed with PBS (pH 7.4) before applying coverslips. The entire smear of each slide was examined using an epifluorescent microscope and oocysts were counted. A positive control slide was used to ensure IFA results. The number of oocysts was expressed per 100 L of filtered water.

### *Cryptosporidium* oocysts genotyping

#### DNA extraction

*Cryptosporidium* DNA was extracted using NucliSENS EasyMag kit (Biomérieux) according to the manufacturer’s instructions. DNA was stored at −80 °C until use.

#### Nested PCR

The *Cryptosporidium* 18S rRNA gene was amplified by a nested PCR assay. In order to highlight the presence of multiple species of *Cryptosporidium* in a single sample, five replicates of nested PCR were performed using different DNA volumes for the first round (10, 2, 1 and 0.5 μl), as reported elsewhere [22]. The first PCR round was performed with primer pair CpXiaoExtF (5′-TTC TAG AGC TAA TAC ATG CG-3′) and CpXiaoExtR (5′-CCC ATT TCC TTC GAA ACA GGA-3′) and the second PCR round was performed with primer pair CpXiaoIntF (5′-GGA AGG GTT GTA TTT ATT AGA TAA AG-3′) and CpXiaoIntR (5′-AAG GAG TAA GGA ACA ACC TCC A-3′). The two PCR rounds were performed under the same conditions. Nested PCR mixtures contained 1× PCR buffer, 5 mM MgCl_2_, 200 μM each deoxynucleoside triphosphate, 100 nM each primer and 1.25 U HotStart Taq polymerase. Cycling conditions consisting of a hot start at 94 °C for 3 min followed by 35 cycles with denaturation at 94 °C for 45 s, annealing at 55 °C for 45 s, and extension at 72 °C for 1 min, and a final extension at 72 °C for 7 min, as previously described elsewhere [30, 31].

#### Restriction fragment length polymorphism

A RFLP assay was performed with two restriction enzymes, *AseI* and *SSpI,* according to the manufacturer’s instructions (Ozyme, Saint Quentin en Yvelines, France). Part of the second-round PCR products was digested with *AseI* and another part with *SSpI*, allowing the detection of different species when present [30]. Restriction profiles were visualized by electrophoresis of digested products on a 2% agarose gel with ethidium bromide.

#### DNA sequencing

Second-round positive PCR products were first purified by ExoSAP-IT^®^ (USB^®^ Products Affymetrix, Inc., Ohio, USA) and bidirectional sequencing analysis was carried out with a 3500xL Dx Genetic Analyser (Applied Biosystems^®^, Life Technologies) using Big Dye Terminator Cycle Sequencing and Purification Kit, according to the manufacturer’s instructions (Applied Biosystems^®^, Life Technologies). Electropherograms were analysed and consensus sequences were constructed using DNA Baser Software. Blast software was used to determine homologies of these consensus sequences with those deposited in GenBank.

## Results

During the surveys, a total of 16 water samples (12 in Port-au-Prince and 4 in Cap Haïtien) were collected from different sites supplying the majority of the population via various modalities of distribution (reservoirs, bores, springs and surface water). Detailed characteristics of sampling sites are presented in Table 1. As shown in Table 1, the IMS-IFA method demonstrated contamination of all water samples (100%) collected in Port-au-Prince and Cap Haïtien by *Cryptosporidium* oocysts with concentrations ranging from 6/100 L (site No. 10 in Port-au-Prince) to 6088/100 L (site No. 13 in Cap Haïtien). PCR detection of *Cryptosporidium* oocyst DNA was successful in 15 of the 16 samples analysed and confirmed 94% of the IMS-IFA results.

Under our working conditions, repeated nested PCR-RFLP using 10 μl of DNA extracts for species identification demonstrated the presence of *Cryptosporidium parvum* in all 15 water samples (Table 1). However, positive PCR results were obtained for four samples (No. 13, 14, 15, 16) when using 2, 1 and 0.5 μl samples of DNA extracts. Species identification was confirmed by direct DNA sequencing, as analysis of electropherograms failed to demonstrate any species diversity in the samples studied. Blast results showed a 100% identity of our sequences with *C. parvum* 18S ribosomal rRNA gene sequences available in GenBank.

## Discussion

*Cryptosporidium* is a strict intracellular parasite and its infectious forms are oocysts eliminated in the stools of various hosts, including humans. Domestic and wild animals are important reservoirs in the transmission of this zoonosis to humans, and water plays an important role in the spread of contaminating oocysts, which are excreted in a variety of environments [6, 10]. Oocysts can survive for long periods in fresh water and are resistant to water chlorination processes. Correct identification of this pathogen is extremely important, not only because of the clinical implications, but also for epidemiological studies. In developing countries such as Haiti, *Cryptosporidium* infection represents a major public health threat. Several factors facilitate transmission of *Cryptosporidium* and account for the propensity to cause large-scale outbreaks of diarrhoea such as those observed in developed countries: (i) *Cryptosporidium* can infect many mammalian species and is frequently identified in farm animals, particularly calves, and in domestic animals; (ii) oocysts are very resistant and can conserve their infectivity in moist environments for a long time; (iii) the genus *Cryptosporidium* is composed of a large number of species, several of which can infect humans; (iv) the infectious dose is very low, and infected individuals excrete large numbers of oocysts, up to 10^8^ in a single day [7].

This study demonstrated the very poor biological quality of drinking water in the two cities. Although only a limited number of water samples were collected in Cap Haïtien, these samples were the most heavily contaminated, probably due to the large number of stock farms located around sampling sites. Exposure to such concentrations of *Cryptosporidium* oocysts in drinking water could generate major biological risks for human health, particularly for children under the age of 5 years, undernourished individuals and immunocompromised patients. Greater commitment to environmental improvement in these cities is therefore required to improve the quality of drinking water in order to limit human transmission of cryptosporidiosis. This is particularly important for the safety of young children and HIV/AIDS individuals. The results concerning the high level of *Cryptosporidium* oocyst contamination were not surprising, as previous studies conducted in Port-au-Prince led to similar results [4]. However, a possible impact of the January 2010 earthquake on these results cannot be excluded, as the study was conducted after the earthquake and the subsequent breakdown in infrastructures damaged a large part of the water reservoirs and water distribution network. This damage may have worsened pre-existing water pollution by *Cryptosporidium* oocysts. Moreover, the presence of *Cryptosporidium* oocysts in water samples from bores ranging in depth from 60 to 106 m raises a number of questions. How were oocysts transferred from the surface to such depths? Damaged pipelines used for water distribution would not explain oocysts contamination, as water samples were collected from bores. The geological characteristics of the ground may explain transfer of *Cryptosporidium* oocysts to groundwater, as Haiti is mostly mountainous and these mountains are mainly limestone, although some volcanic formations can be found, particularly in the Massif du Nord. Karstic features, such as limestone caves, grottoes, and subterranean rivers, are therefore present in many parts of the country, including the Cul-de-Sac Plain aquifer that constitutes the main source of drinking water for the urban population of Port-au-Prince. Karst aquifers are known to be highly susceptible to microbiological pollution [11, 17, 18].

The other objective of this study was to identify *Cryptosporidium* species from oocysts collected in water samples. Although PCR is extremely sensitive and efficient when a pure DNA suspension is used, amplification can be reduced by inhibitory factors frequently present in environmental samples, which could be a possible explanation for the unsuccessful PCR assay for sample No. 7 despite the positive IMS-IFA result. Contrary to the experimental results of Ruecker’s study in 2011 [26], PCR assays performed with DNA extract volumes < 10 μl were less successful for our environmental samples, as these assays were positive for only four samples (No. 13, 14, 15, 16) containing the highest loads of *Cryptosporidium* oocysts (Table 1). The success of this method therefore depends on the presence of a sufficient quantity of *Cryptosporidium* DNA before dilution. In Haiti, *Cryptosporidium* species identification has been previously performed on the stools of patients with cryptosporidiosis, but never on environmental samples. Under our working conditions, using both genus-specific nested PCR-RFLP analysis and direct DNA sequencing, only *Cryptosporidium parvum* was identified in the water samples studied (Table 1). This result was somewhat surprising, as a diversity of *Cryptosporidium* species was expected in view of the insalubrious environment in the two cities. Practically no tests are performed in Haiti to ensure the quality of the water distributed by public services. In urban areas, basic services for collection and treatment of wastewater and solid wastes including sewerage are not available. Latrines and septic tanks result in faecal contamination of alluvial and karstic aquifers [9]. Moreover, animals are allowed to roam freely in the cities and scatter their stools everywhere. Although animals have not yet been evaluated as a possible reservoir for *Cryptosporidium* in Haiti, the close proximity between Haitians and both domestic and wild animals may affect the rate of *Cryptosporidium* infection, as a study conducted in Port-au-Prince between 2000 and 2001 based on 1,529 stool sample examinations revealed the presence of *Cryptosporidium* oocysts in 158 (10.3%) stool samples [24]. In this same study, genotyping of 69 *Cryptosporidium* oocysts demonstrated the presence of three *Cryptosporidium* species: *C. hominis* (41), *C. parvum* (26) and *C. felis* (2). Poor resolution of possible mixed species could therefore be due to the methodology used. However, the performance of nested PCR-RFLP analysis for *Cryptosporidium* genotyping has been clearly established [8]. More recently, the performance of repeated nested PCR-RFLP, as used in our study, was assessed for its ability to detect mixtures of *Cryptosporidium* species in water samples [26]. Furthermore, it had been reported that direct sequencing of PCR products is more accurate than cloning to characterize *Cryptosporidium* species in water [27]. Despite the use of these two methods, only *C. parvum* was found in the water samples analysed in the present study. Either these samples effectively contained a single species, or they contained a mixture of species that was not detected by our methods. A possible explanation could be that *C. parvum* was the predominant species present, preventing identification of the less abundant species by molecular methods. The results of this study therefore cannot exclude the presence of other less abundant *Cryptosporidium* species in the water samples analysed, as most of the roaming animals in the cities of the study were pigs, oxen, goats and donkeys, which could be the main sources of *C. parvum* oocysts in the environment. However, this explanation would appear to be in disagreement with the results of a previous study showing that *C. hominis* was the leading cause of human cryptosporidiosis in Haiti [24]. Human-to-human transmission of *C. hominis*, the species most commonly responsible for human infection, would therefore appear to be frequent. However, a study conducted in the same area in 2006 revealed that human-to-human transmission appeared to be an unlikely route of infection, as among 123 persons living in close contact with 56 HIV-positive subjects contaminated by *Cryptosporidium*, only 1 was infected [25].

## Conclusion

This study confirmed *Cryptosporidium* oocysts pollution of drinking water in the study sites, probably worsened by the January 2010 earthquake, but it failed to reveal the expected species diversity of *Cryptosporidium*. Nevertheless, the study constitutes the first molecular characterization of *Cryptosporidium* oocysts conducted on environmental samples in Haiti and it also highlights the difficulty of detecting mixed populations of *Cryptosporidium* species on environmental samples.
